# Toward Tailored and Targeted Communication for the Promotion of Firearm Safety: A Qualitative Study With Firearm Retailers

**DOI:** 10.1177/00332941241256880

**Published:** 2024-05-31

**Authors:** Mike Henson-Garcia, Lauren Q. Malthaner, Katelyn K. Jetelina, Michael Mackert, Marlyn Allicock, Sandra McKay

**Affiliations:** Department of Health Promotion and Behavioral Sciences, 12340 University of Texas Health Science Center at Houston (UTHealth Houston), School of Public Health, Dallas Regional Campus, Dallas, TX, USA; McGovern Medical School, 12339 University of Texas Health Science Center at Houston (UTHealth Houston), Houston, TX, USA; Department of Epidemiology, Human Genetics, and Environmental Sciences, 12340 University of Texas Health Science Center at Houston (UTHealth Houston), School of Public Health, Dallas Regional Campus, Dallas, TX, USA; The Stan Richards School of Advertising and Public Relations, 12330 University of Texas at Austin, Austin, TX, USA; Department of Health Promotion and Behavioral Sciences, 12340 University of Texas Health Science Center at Houston (UTHealth Houston), School of Public Health, Dallas Regional Campus, Dallas, TX, USA; McGovern Medical School, 12339 University of Texas Health Science Center at Houston (UTHealth Houston), Houston, TX, USA

**Keywords:** Audience segmentation, targeting, tailoring, firearm safety, gun shop, firearm retailer

## Abstract

Firearm injury is a major yet understudied public health issue in the U.S. This qualitative study explored firearm retailers’ perspectives to inform messaging and communication approaches to promote firearm safety among the gun owning population. Semi-structured interviews were conducted with 17 retailers at a single gun shop in Texas. Thematic analysis identified key themes related to (1) audience segmentation, (2) appropriate use of language, and (3) trusted messengers and modalities for the communication of firearm safety information. This formative work provides practical insights to optimize public health messaging in this arena and ultimately reduce firearm injuries. Overall, this study provides valuable insights to guide the development and implementation of evidence-based, social marketing efforts aiming to promote firearm safety across various gun-owning audiences.

Scholars have long recognized firearm violence as a multifaceted yet preventable problem warranting a public health response to elicit meaningful change (Baroni & Richmond, 2006; Bulger et al., 2019; Hemenway & Miller, 2013; Patel et al., 2022; Sinauer et al., 1996). However, the passage of the Dickey Amendment in 1996 – barring the Centers for Disease Control from allocating funding to research projects that promoted the idea of “gun control” — has severely curtailed a generation of public health scholarship in this field. Ultimately, this has hindered progress in mitigating the deleterious impact of firearms on the health of the public (Ault, 2021; Rostron, 2018). Today, firearms are responsible for over 48,000 lives lost in the United States (Centers for Disease Control and Prevention, 2023) and has been estimated to cost the nation over $4.2 trillion (Peterson, 2021).

As with other consumer products that can be injurious to health and wellbeing, such as tobacco, alcohol, and motor vehicles, the application of *social marketing* paradigms may yield beneficial results for the promotion of firearm safety and, in turn, the prevention of firearm-related injury. In fact, a systematic review of 54 health promotion campaigns and interventions found that the application of the core tenets of social marketing to modify behavior was enormously effective in producing desired behavioral change across a plethora of health issues, target audiences, and settings (Stead et al., 2007). Social marketing has been a topic of interest among public health scholars for many years, with various definitions being proposed. However, the definition put forth by Grier and Bryant (2005) has gained widespread recognition and acceptance in the field. According to their conceptualization, the primary objective of social marketing is to use “commercial marketing concepts and techniques” to influence the attitudes, norms, and behaviors of individuals or groups, referred to as the *target audience*, with respect to specific health or social issues. Grier and Bryant (2005) emphasize that social marketing is not just about promoting healthy behaviors but also about encouraging the abandonment or modification of harmful ones.

To successfully apply this approach to the promotion of firearm safety, investigators must first engage in formative research to gain a deep understanding of the target population’s needs, values, aspirations, motivations, attitudes, and norms. This research is critical to the success of social marketing as it allows for the segmentation of the larger target population into distinct subgroups. This process is known as *audience segmentation* and represents a key strategy used by health communicators in social marketing activities (Slater, 1996). The development of effective social marketing campaigns and health promotion interventions often relies on understanding the unique characteristics, behaviors, and motivations of various audience segments within a population. By designing and tailoring messages that resonate with each audience, health communicators can create more impactful campaigns. In fact, health communication efforts that fail to segment larger populations into distinct sub-audiences are thought to be less effective in attaining their intended outcomes (Noar et al., 2007). Additionally, scholars have argued that communication techniques and behavioral interventions that are targeted, tailored, and personalized for specific sub-audiences are more efficient and cost effective than one-size-fits-all, universal approaches (Altendorf et al., 2022; Ishikawa et al., 2012; Kreuter et al., 1999; Mackert et al., 2021; Teeny et al., 2021; Zhou et al., 2020). Despite the proven cost-effectiveness and efficacy of audience-centered interventions and campaigns in promoting behavior change, there is a notable scarcity of research investigating the most effective strategies for applying these approaches to encourage the adoption of firearm safety behaviors among varying gun-owning audiences.

Identifying shared characteristics among audience segments can serve as a catalyst for developing impactful messaging strategies in a cost effective manner. To this end, some empirical research has begun to reveal the considerable heterogeneity that exists among the gun-owning population in the United States, emphasizing the importance of tailoring communication strategies to effectively reach sub-audiences of the firearm-owning population in the United States. For instance, Kalesan et al. (2016) were some of the first to empirically demonstrate that participation in a social gun culture was strongly linked to gun ownership and immense regional variation in the expression of these gun cultures tend to occur across the US (Boine et al., 2020). Additionally, Boine et al. (2022) found that six distinct subgroups exist among the American gun-owning population including (1) the family protectors, (2) incidental gun owners, (3) the Second Amendment activists, (4) the target shooters, (5) the hunters, and (6) the self-protectors. In another study exploring patterns of firearm storage beliefs and firearm-related risk, three distinct profiles emerged among gun owners: one group asserting that guns ensure safety within the home environment and should be made readily accessible, one group believing the safety provided by keeping a firearm stowed in the household firearm is highly context-dependent, and a final group that perceived no risk whatsoever when firearms are stored appropriately in the home (Salhi et al., 2021). Altogether, the accumulating evidence indicates that the gun-owning population in the United States is not a monolith. Consequently, there is an urgent need to comprehend how to optimally develop tailored and targeted campaigns and interventions that will resonate with the different types of firearm owners that are thought to exist within this rapidly growing population.

Firearm retailers, along with other professionals who interact with and utilize firearms as part of their job duties (i.e., military veterans, law enforcement officers), have been consistently identified as trusted sources of firearm safety information among various samples of firearm owners (Aitken et al., 2020; Anestis et al., 2021; Crifasi et al., 2018; Ewell Foster et al., 2024). Given their status as widely trusted messengers, our goal was to learn the best ways to communicate with firearm-owning audiences and examine their informational needs by focusing on interviewing firearm retailers. This decision was based on two key factors. First, firearm retailers are considered to be uniquely positioned to build community trust and often believe that integrating firearm safety programming in the retail space is “an important service to [their] community” (Barnard et al., 2022; Polzer et al., 2021). Second, recent research has shown a decline in public trust toward both the US military and law enforcement institutions (Pew Research Center, 2022, 2023), making firearm retailers a more suitable choice for this study. Therefore, the purpose of this qualitative study was to delve into the perspectives and experiences of a group of firearm retail employees – a pivotal set of stakeholders that regularly interact and communicate with firearm owners – to identify potentially efficacious strategies and approaches for tailoring and targeting communications related to firearms and firearm safety.

## Methods

### Study Population

Semi-structured interviews were conducted with 17 firearm retail associates employed at a medium-sized firearm shop and range located in the suburbs of Houston, Texas between June 2022 and August 2022. Almost all participants were male (*n*=16/17). Interviewees played diverse roles at the gun shop, encompassing a broad spectrum of responsibilities, including retail associates, range officers, armorers, firearm safety instructors, member services managers, and upper management.

### Data Collection

Several site visits were made by the principal investigator on the project (SM) where participants were informed about the study and recruited for participation. Timing of visits were varied on day, evening, and weekend over two months to capture diversity in the staff working at the retail location. Study purpose and expectations were reviewed and informed consent was obtained prior to enrollment. Recruitment of these retailers occurred via a purposive sampling technique ref. One member from the research team (MHG) — a male, Hispanic doctoral student with expertise in health communication, health promotion, and the behavioral sciences — conducted all interviews with participants online utilizing the WebEx virtual conferencing software. Interviews ranged from 40 minutes to 1.5 hours. All participants received a gift card for their participation in the research study. The Committee for the Protection of Human Subjects at the University of Texas Houston Health Science Center reviewed and approved this study (IRB# HSC-MS-22-0002).

### Theoretical Frameworks

In this qualitative study, using existing literature and expertise of the team, we developed a semi-structured interview guide, containing 25 questions and accompanying probing questions, to explore retailers’ perspectives on the development and implementation of firearm safety communication interventions within the firearm retail and range environment. The interview guide covered a range of topics beginning with an examination the retailers’ job responsibilities and interactions with customers, followed by inquiries on the provision and communication of firearm safety information, the influence of customer attributes on the provision of safety information, and thoughts on effective and popular firearm safety devices. The second portion of the guide solicited retailers’ opinions on a proposed initiative involving point-of-sale firearm safety information and counseling. The guide also incorporated questions assessing retailers’ attitudes and normative beliefs regarding firearm safety efforts, as well as their self-efficacy beliefs concerning their participation in such endeavors. This section aimed to capture information about the possible implementation of the intervention within the retail environment, providing valuable information for potential program planners, as well as addressing required resources and organizational support, potential advantages and obstacles of the intervention, and the perceived influence of the intervention on firearm-related health outcomes, such as unintentional injuries among youth, suicide, and crime and homicide. Lastly, the guide examined retailers’ perceptions of the program’s potential sustainability and effectiveness.

Ultimately, the questions in the interview guide were informed by Theory of Planned Behavior (Ajzen, 1991), the implementation outcomes framework (Proctor et al., 2011), and core concepts from social marketing theory (Hastings & Saren, 2003). Table 1 details the ways in which these theoretical constructs and principles map onto the interview questions employed in the present study.

**Table 1.** Semi-structured Interview Questions With Firearm Retail Employees.

Theory	Construct	Interview Question(s)
Theory of planned behavior	Attitudes toward firearm safety	• Could you tell me your thoughts, opinions, and feelings about firearm safety?
		• Do you think firearm safety is an important issue? Why or why not?
		• Are there issues that are more important than firearm safety? If so, why, and what are they?
	Normative beliefs toward firearm safety	• Do you think the way we commonly think about firearm safety needs to change? If so, how?
	Self-efficacy in providing firearm safety information	• Could you describe how confident you feel in providing a customer with firearm safety information at this very moment?
		• What would help you feel more confident to do something like this if anything?
Implementation outcomes framework	Acceptability of firearm safety counseling interventions	• What are your general thoughts about [delivering firearm safety information to customers at the point of sale]?
		• What are the components that would work well in the retail/range environment?
		• What are the components that wouldn’t work so well in the retail/range environment?
	Appropriateness of firearm safety counseling interventions	• What do you think your customers would think about firearm safety counseling at the point-of-sale?
		• In other words, how do you think your customers would feel about being given firearm safety information during their transaction?
		• Do you think your customer base would be receptive to firearm safety information? Why or why not?
	Implementation costs related to firearm safety counseling interventions	• What are some potential benefits of firearm safety counseling at the point-of-sale?
		• What are some barriers or challenges you can think of related to firearm safety counseling at the point-of-sale?
		• What might your organization need to start providing firearm safety counseling at point of purchase?
		• Are there resources that your organization would need to do a program like this? If so, could you tell me what these would be?
		• Are there resources that you or your organization currently possess that would make this program a success to your organization? If so, what are these?
	Feasibility and impact of delivering firearm safety counseling interventions	• Do you think it will be challenging to integrate firearm safety counseling into your job duties or the duties of employees at your store? Why or why not?
		• From a business perspective, what would be the important factors to consider when integrating firearm safety counseling at the point of sale?
		• How might the integration of firearm safety counseling at point-of-sale interfere with business if at all?
		• What impact, if any, do you think this idea has in your community?
		• Do you think firearm safety counseling would be an effective strategy in preventing firearm-related accidents? What about in preventing suicides? What about in preventing crime?
	Sustainability of firearm safety counseling interventions	• Do you think providing firearm safety counseling at the point-of-sale is a sustainable effort? Why or why not?
		• What, in your opinion, are the important things that are needed to ensure an effort like this would be maintained over time?
		• Do you think this program, if implemented, would be able to keep running for a long period of time?
Social marketing principles	Audience segmentation	• Thinking a little bit more about your interactions with customers, do you ever provide firearm safety information?
		• Does the type of customer (appearance, gender, age, etc.) influence the type of information you provide on safety?
	Tailoring information	• What type of firearm safety information do you give out to customers?
		• Who are the sources or authors of the information you give out?
		• Do you think the materials are attractive or persuasive? Why or why not?
		• Do you provide information related to suicide prevention? What about information related to accident prevention?
		• Do you think there is a need for providing this information to customers?
	Message placement and promotion	• How do you think this method of giving out information compares to other options (like word-of-mouth, posters, websites, or phone apps)?

### Qualitative Analyses

For this study, two authors (MHG & LQM) utilized NVivo software to code all interview transcripts using a deductive codebook created a priori to assist with coding, supplemented by inductive codes for any emerging themes (Braun & Clarke, 2006). To establish interrater reliability, coders met to resolve any discrepancies, and percent agreement ranged from 83.93% to 100%. The development of the codebook followed an iterative process that involved the creation and application of both deductive and inductive codes. Deductive codes were derived from the constructs of the theoretical models guiding the study, while inductive codes emerged through the use of descriptive coding techniques. These codes were then systematically applied to the data and subsequently categorized into broader themes to facilitate analysis and interpretation.

### Positionality Statement

The research team comprised two doctoral-level graduate students and four professors, each possessing expertise in health promotion planning, behavioral sciences, health communication, pediatrics, and injury and violence epidemiology. The team consisted of two male and four female researchers, each with diverse experiences related to firearms and firearm injuries.

One researcher (MHG), a doctoral student focusing on firearm injury prevention and health communication, has had family members and close friends who were victims of firearm homicide and suicide. The second author (LQM), an expert in injury and violence prevention epidemiology, neither owns a firearm nor has personal experience with firearm injury. Another author (KKJ), an injury prevention epidemiologist and firearm owner, has a spouse working in law enforcement. A fourth author (MM) has lost a family member to firearm-related suicide and is an expert in marketing, advertising, and health communication. The fifth author of this work (MA), a behavioral scientist and expert in qualitative methodology, neither owns a firearm nor has personal experience with firearm injury. Lastly, the final author (SM), a pediatrician and firearm injury prevention advocate, is a firearm owner with relatives in law enforcement and provides medical care for victims of firearm injury.

Despite the team members’ varied experiences with firearm injury and firearm culture, throughout the research process, the authors remained committed to engaging in reflexivity to minimize the potential influence of personal biases on the reporting and dissemination of the study findings. All authors contributed to every phase of the research, including conceptualization, data collection and management, data analysis, and manuscript writing.

## Results

In this paper, we present insights gained from interviews with retailers regarding the demographic characteristics of firearms consumers who need more information on firearm safety. Additionally, we also focus on the common communication strategies employed by retailers in this setting. Related to this work, thematic analysis revealed three major themes (i.e., (1) insights surrounding pertinent audience segments, (2) the language of firearms and firearm safety, and (3) preferred messengers, modes, and communication channels) accompanied by several subthemes within each theme. Representative quotes for all themes and subthemes can be found in Table 2.

**Table 2.** Representative Quotes for Insights on Communicating Firearm Safety.

Parent theme	Sub themes	Representative quotes
Audience segmentation insights	First-time firearm owners	“You can kind of tell if the person buying the firearm is new, and at that point we will offer firearm training. You’ll meet your regulars that know what they’re doing. So there’s no, you know, tell them what to do. That would annoy them. But you can tell the new shooters from the experienced.” (participant 7, retail associate and range)
		“The threshold is where you have to stay and it becomes a challenge. When you become – when you’re dealing with people that have been around firearms their whole lives and you’re treating them like a beginner that threshold is difficult because there’s a tolerance there that they’re only going to tolerate so much.” (participant 8, lead instructor and director of ranger operations)
		“Even if they’ve been shooting all their lives, most of the ones that have been shooting all their lives that I’ve seen and dealt with have safes, actual gun safes that you can’t break intact too easily. New firearm owners have a gun safe, but it’s one of the inexpensive ones because that’s what they can afford at the time.” (participant 11, manager)
	Firearm-owning families	“And the biggest thing with kids… is making sure the parents are in tune with what’s going on. ‘Cause the parents can lay in the secondary and bring in more information to the kids and be open to the kids, 'cause the kids are going to have questions. And be open to them and help them.” (participant 8, lead instructor and director of range operations)
		“Usually when it comes to kids and being around firearms at home, that overall responsibility of the firearm is up to their parents or whoever, mom or dad, whoever owns the firearm. That is the job and responsibility to make sure that it’s put up and away from kids, whether it be in a safe, up somewhere high to where they can’t get it or even know about it.” (participant 9, range officer and NRA-certified instructor)
		“If you’re trying to target the people who are owning firearms to protect their homes, but they also want to protect their children, or elderly, or other loved ones who maybe aren’t very safe around firearms, there’s I think probably a different messaging. Because no one’s going to keep their firearm unloaded if the whole reason they got a firearm was to keep it loaded at the house” (participant 21, new firearm owner)
	Military and veteran populations	“We do have a few flyers up around the store, where it’s if you need help. Because we have such a large veterans community… that come in and suicide among veterans is just through the roof right now.” (participant 6, hospitality director)
		“I’ve seen plenty of military and law enforcement guys that have no idea what they’re talking about, even though they’re in that job or profession around firearms. You know what I’m saying? I Would say it kind of just depends on everyone’s personal background history and what their previous experience might be.” (participant 9, range officer and NRA-certified instructor)
		“Well, in most gun stores, I would say or firearm retailers, most of them have military veterans who work there. Now I know that sounds cool but not every military veteran knows how to operate a gun. I Know that’s surprising. But just because you have some kind of military background doesn’t mean that you are an expert in the field of firearms because not everybody has to kick a door in and kill bad guys in the army. There’s people who drive trucks. There’s people who push paperwork.” (participant 10, retail associate and armorer)
	Female gun owners	“Women have a lot steadier hands than men, too, so they really receive a lot of information better when it has to do with firearms, ‘cause I think women have more fear in them than men do when it comes to firearms, which is a good thing, 'cause we should be scared of guns.” (participant 5, retail associate and range officer)
		“Women are a whole lot better at saying I don’t, I don’t know anything about this. So a lot of that comes out in the one, on one type of training class when the trainer can actually identify the weaknesses in the customer’s knowledge of the firearms and the safe working and safe keeping of the firearm.” (participant 12, director of compliance and procurement)
		“Really, we want to be family with everybody that comes in, welcoming to women … we don’t want to be intimidating like you would think a gun store would be.” (participant 16, general manager)
Language considerations	Appropriate usage and connotations of the terms *firearm* versus *gun* versus *weapon*	“So if people are being told about gun safety – and this is just maybe kind of my opinion – but it could sound informal and maybe not as professional or backed by professionalism. Whereas if I’m talking to a customer about firearm safety, then that sounds more professional. It sounds more formal, and like something to be taken more seriously than gun stuff.” (participant 4, retail associate)
		“So the right terminology, we just say firearm, handgun, rifle, firearm, something that doesn’t put that stigma of saying like, “this is a dangerous thing.” because the firearm itself is not a dangerous thing. It’s the person behind it, using it.” (participant 11, manager)
		“For the firearm: So a lot of people get scared when you say “gun” or “weapon.” so at our store we refrain from using those terms and instead call it “firearm.” but all three terms are correct: Gun, weapon, firearm – they’re all correct. Sometimes for the customers, whenever I’m teaching a class and they’re kind of waving the gun or they point it at me, I say, “hey, you’re pointing a weapon at me,” because it didn’t connect in their mind that this firearm is dangerous. So whenever you say “weapon” or “gun,” that’s whenever it connects into their mind of, “oh, that’s right; this is dangerous.”” (Participant 15, retail associate)
	Misuses of terminology related to firearms	“We don’t use the term assault rifle. This is not an assault rifle. This is an AR platform rifle, or firearm, whatever it is… the AR does not stand for assault rifle. It stands for ArmaLite rifle, which is an actual company, and we’ll kind of go through a little bit of the history with the AR platform, things like that.” (participant 1, general manager)
		“I will say that sometimes the term like, ‘assault weapon’ or ‘assault rifle’ kinda gets thrown around a lot, and I think that kind of maybe puts like a negative connotation on some weapons. People may feel like if something is being called an “assault weapon” or something like that, then they may not get like a really good understanding of what that tool is used for, or what its potential is. And there’s not really a clear definition of what maybe an assault weapon is.” (participant 4, retail associate)
		“I mean, a firearm in and of itself is not an assault weapon. And in fact, assault rifle isn’t even - no civilian can own an assault rifle, right? AR doesn’t mean assault rifle. It means ArmaLite rifle. And it was named after the company who first made it. So, you know, that’s part of that whole education stuff where that the gun haters don’t know what they’re talking about and they spew misinformation. (Participant 12, director of compliance and procurement)”
	Analogies used to communicate firearm safety information	“I mean, essentially, this gun is no dangerous than a hammer or a screwdriver… a tool, just like you use any other tool to help you achieve a task, whether it be for self-defense or just for shooting on the range, having a good time, whatever it is…” (participant 1, general manager)
		“Everybody thinks that guns kill people, and it’s not true. It’s just like a car, right? It’s not the car that killed the person, it’s the person behind the wheel. If you’re not driving safe or you’re driving drunk, who do you blame it on? The car doesn’t get charged? Ford doesn’t get charged. It’s the person. It’s the same thing with guns.” (participant 2, NFA manager)
		“In my opinion there is no difference between a screwdriver, a hammer, and a firearm. If you miss a screwdriver and you mishandle a screwdriver then you get a gouge or a hole in your hand from the screwdriver, you slipped and you hurt yourself. If you miss the nail you hit your thumb and you have a sore thumb. With a firearm you’re dealing with a projectile coming out at 1200 feet per second, that’s four football fields in one second, so it’s traveling at a velocity that would easily destroy something. (Participant 8, lead instructor and director of ranger operations)”
Messengers, mediums, and communication channels	Firearm retailers as credible and trustworthy messengers	“Yeah, I definitely think I’m the right person. I mean, if I’m the one selling you a gun, then I need to make sure you walk out of the store with all the knowledge I can put in your brain, right? I mean, who else better to do it than the person that is helping you at that time, that’s helping you with training, that’s helping you buy a firearm. That’s kind of part of the job. I don’t just sell you a gun and let you walk away. I Make sure you have everything you need from ammo to cleaning equipment, and then also knowledge, as much as I can give you. I Think there’s nobody better than that person you’re buying the gun from.” (participant 2, NFA manager)”
		“Oh, absolutely. I think, again, that’s how you build rapport. That’s how you build an at-ease customer. It exudes and shows that you care about the community, you care about safety, you care about firearms, and you care about them. And I think that’s the real opportunity. That’s the binding thread of anybody who comes into a gun range, is talking to them about safety, ‘cause it shows that you can be empathetic if they’ve had a bad situation, you’re educating, you’re not there just here to buy a gun from me and whatever you do with it is up to you. (Participant 8, lead instructor and director of ranger operations)”
		“I don’t think there would be anybody else, other than the… people who are selling the firearms, selling the product…. You can’t really go talk to the manufacturers of the firearms. I’m sure that they — of course they probably have [safety information] on their websites, and stuff like that, but it’s the human to human interaction that really is the most important thing… so yeah, I think gun retailers are the only place that this should be done at.” (participant 16, general manager)
	Lack of a structured communication delivery system	“If people have questions about firearms, “okay, I’m going to go to someone who knows about firearms. Who is that? Oh, here’s a gun club. They know what they’re talking about, so I’m going to listen to them.” now, I will say that is not the standard across the board… You can go to academy, a big firearms retailer, and they’ve got some 18-year-old kid behind the counter. “Hey, can you tell me about this gun?” “It’s a gun. It has a trigger, you pull it, and it goes BANG.” It’s not that something like [firearm safety counseling in the retail environment] couldn’t be implemented, but how likely would it be to have that implemented at the same level across the board?” (participant 1, general manager)
		“Like when customers have questions about safety, or if we’re just kind of talking to them about how they plan to carry, or how they plan to store their guns. I Think it would be good to have maybe a more formal, structured way of doing that with every customer, and maybe instead of just more with the customers that didn’t ask more about safety and proper storage and stuff.” (participant 4, retail associate and range officer)
		“In the retail space I think it’s just an overall conversation, to be honest with you. I don’t have a way to implement [conversations] in a formula every time, because you just have such a vast assortment and range of experiences of people coming into a gun range.” (participant 8, lead instructor and director of ranger operations)
	Differentiated safety information based on situation and context	“If someone’s planning on having a gun in their car for self-defense but not on their person, I recommend having it in a locked place, like a glove compartment. Or sometimes what I’ll recommend is, people can have a lockbox maybe under their driver’s seats that also somehow attaches to the car in some way, so if the car is broken into, someone can’t just grab the lockbox and take it.” (participant 4, retail associate and range officer)
		“So whenever they’re buying a gun, if it’s their first time buying a gun, I’ll ask them what they’re using it for, and I kind of give them just some safety tips, necessarily. If they’re using it for home defense and they have kids in the house, make sure it’s up high in an unreachable place or locked in a – the best way I say it is in a biometric safe… if they don’t have children, basically tell them it’s your own discretion if you wanna keep the magazine separate from the gun. Just to avoid any of that, you can, but people also have to remember that on situational stuff that it's split-second decision timing, so that is what I tell them.” (participant 5, retail associate and range officer)
		“But I believe that if you have a child in the house or I think just children in general, you should get some kind of safe because hiding a gun doesn’t really do much. Children are curious. They will find it eventually. And then if they don’t know how it operates or when it does, then somebody could get hurt.” (participant 10, retail associate and armorer)
	Utilizing pamphlets to communicate firearm safety	“I think that it would be good to have visual components, because you may not always be dealing with a customer that maybe like understands fluent English, for instance. But we still wanna get the concept across, like basic gun safety, how you wanna store it safely and stuff. It’d be good to have some kind of visual aid in doing that, so that people can still understand, regardless of like their language fluency or I guess maybe even like literacy. Because literacy is still a thing.” (participant 4, retail associate and range officer)
		“The language barrier for sure. We get a lot of people who don’t speak English very well. That’s something to think about.” (participant 16, general manager)
	Utilizing posters to communicate firearm safety	“Right before you walk onto the range, right when you get to the retail counter, we have posters that are everywhere. Now, that is assuming that people will read those rules. And the thing is that I mean, to be honest, half the time people walk in here asking for the restroom and the restroom is right to their left. It’s right there and people just refuse to look at it. Whether it’s their first time or not, I mean, I don’t blame them, but I don’t think posters are the way to go.” (participant 3, retail associate and range officer)
		“And for me, what I’ve realized is people don’t stop and read, most people don’t stop and read or look at posters or signs. It really has to get their attention or they have to be looking for it. People will just walk by it.” (participant 16, general manager)
	Utilizing technology to communicate firearm safety	I mean, imagine if you were on instagram or on facebook or the kids, if they’re on tik-tok, and there were… huge capital investment push behind creating great content about firearm safety. Right?.. What if we used some of this stuff and really communicated? What if we created an age-appropriate content class for younger kids and was in ranges and then there was a big social media push where they’re going to see it, and they go to these ranges and participate in this, you know one-day program and have that as a resource? What if, you know, xbox and PlayStation had a little ad on all of their call of Duty’s or whatever the hottest new thing – Apex or whatever the hottest, new shoot-them-up game is, what if they had some kind of ad in the loading screen about this program? You know? So I think that as long as it's generationally appropriate for the means of delivering that information, then I think the technological approach to [delivering firearm safety information] is very smart. (Participant 14, vice president and COO)
		“I’d say [technology] plays a major role, especially in a sense most people have phones with them at all times. They have the internet with them at all times. So I feel like if we have the information out there available to them in a way that it's accessible, again, people who are interested will find their way to it.” (participant 15, retail associate)

### Theme 1: Audience Segmentation Insights

Our analysis revealed four distinct audience segments with regard to communicating firearm safety: (1) individuals who are new firearm purchasers, (2) parents and caregivers who own firearms, (3) veterans and individuals with a military background, and (4) single women with protection motivations. Additionally, participants highlighted the importance of assessing personal background, firearm handling behaviors, and previous shooting experience when evaluating firearm purchasers’ knowledge and proficiency of firearms.

Retailers commonly discussed how they identified new firearm purchasers based on their behavior and actions in the retail environment. For instance, they described how new purchasers may buy less expensive gun safes or may handle firearms in a way that suggests they are not familiar with proper gun safety practices. Additionally, the participants described the varying responses they receive from different customers when they provide firearm instruction. One retailer highlighted the following point:

“...when you're dealing with people that have been around firearms their whole lives and you're treating them like a beginner that threshold is difficult because there's a tolerance there that they're only going to tolerate so much” (male, Lead Instructor and Director of Range Operations)

This observation emphasizes that receptiveness to firearm safety instruction may vary based on an individual’s familiarity and experience with firearms: while new purchasers may be open to learning about gun safety practices, other more established owners might be resistant or fail to fully appreciate the significance of adhering to safety guidelines.

Moreover, participants highlighted the need to communicate firearm safety to gun-owning parents and caregivers. Specifically, retailers suggested that these individuals played a significant role in ensuring that children understand firearm safety and should be open to discussing the topic with them. They also suggested that implementing structured firearm safety education programs in schools could help to reduce firearm violence by dispelling misconceptions and educating young people on the realities of firearms. Additionally, participants suggested that there is a need for tailored messaging about firearm safety to firearm owning parents and caregivers who primarily own firearms to protect their homes and families.

Another potential target audience for consideration is the veteran and military population – which was a segment that was mentioned frequently in conversations with firearm retailers. Firearm retailers identified themselves as credible messengers of suicide prevention information by providing information and resources to customers, particularly those in the veteran population who may be at higher risk for suicide. One employee, who worked as the hospitality director, noted: “…We have such a large veterans community, I guess would be the right word, that come in, and suicide among veterans is just through the roof right now.” Another retailer, a male who served as the Director of Range Operations, differentiated and clarified that they felt confident providing customers with suicide prevention “information” as opposed to “counseling”:

**Retailer:** Are you talking about providing information?

**Interviewer:** Providing information and having conversations with customers about firearm safety. That’s the “counseling” piece, like a back and forth dialogue between customers.

**Retailer:** Oh, absolutely. I think, again, that’s how you build rapport. That’s how you build an at-ease customer. It exudes and shows that you care about the community, you care about safety, you care about firearms, and you care about them.

Participants also indicated that one’s military and law enforcement expertise does not necessarily equate to firearm expertise. For instance, another retailer remarked: “And I come in here every day, and I see police officers shoot, and it's embarrassing. And I understand why we're in the situation we are today. I think police need a lot more training than what they've been held accountable for, and I also think anybody that owns a gun should have some sort of basic two-hour-long safety course before you can even buy a gun.”

Lastly, the role of gender in communicating firearm safety surfaced as a significant theme that emerged in this study. Retailers emphasized the necessity of considering the preferences of female gun owners, who may be less acquainted with firearms or feel intimidated by gun stores. Participants noted that women often engage in specialized training programs and events, such as “Girls Wanna Have Guns” or “A Girl and a Gun.” These initiatives were thought to foster an inclusive and supportive atmosphere for learning about firearms and firearm safety in a way that was tailored for those who identify as female.

### Theme 2: The language of Firearms and Firearm Safety

Firearm retailers strongly suggested being cognizant about language used in communicating about firearms and firearm safety. The majority of retailers endorsed the use of “firearm” and “gun” over “weapon” because these terms are perceived as “less threatening”. Retailers explicitly mentioned avoiding the use of “weapon” to avoid further stigmatize and demonize firearms and to discuss the situational contexts in which using the term weapon is and is not appropriate. For example, one retailer, who served as the NFA manager, reported: “Yeah, I mean we try to stay away from weapon. Firearm, gun, handgun, rifle, shotgun, whatever. We try not to say weapon. You don’t want to put that in somebody’s head, “You’re buying a weapon.” It’s a tool. You use it for a lot of things.” Another retailer discussed the connotations and specificity associated with the use of these different terms: “‘Weapon’ could be a very broad, it doesn't even really always pertain to firearms. It could be some other kinda weapon, a knife or something. ‘Firearm’ is more specific; people know you’re talking about guns when you say ‘firearm,’ but it also maybe sounds a bit more professional.”

Additionally, firearm retailers’ detailed common misuses of language related to firearms and the implications these misnomers can have on influencing perceptions around firearms. Firearm retailers noted that the use of certain terminology, like “assault rifle” or “assault weapon,” fostered inaccurate negative connotations as the terms often get misconstrued when referring to ‘AR’ firearms, or ArmiLite Rifles. Participants underscored the value of instructing customers on the accurate utilization of terminology and definitions, such as differentiating between magazines and clips, and employing the proper names for firearm components. Retailers frequently observed customers employing incorrect terms during point-of-sale discussions. In summary, the participants strongly recommended the use of precise language that faithfully represents the firearm’s function and structure as opposed to resorting to emotive or sensationalized terminology.

Participants also used various analogies when communicating about firearms and firearm safety. Participants commonly drew comparisons between guns and tools to normalize firearms and redirect attention to the user rather than the device itself. For example, they made parallels to household tools like hammers and screwdrivers to assert that when used properly, firearms can be safe, important, and effective tools. By using the tool analogy, participants highlighted the idea that responsible firearm ownership requires a certain level of knowledge and expertise, just like any other tool or piece of equipment. Additionally, many participants made an analogy between firearms and cars to convey the idea that, like cars, firearms can be dangerous if not used properly, but are not inherently dangerous by themselves. These respondents argued that just as a car doesn't cause accidents, but it is the driver who is responsible for driving it safely, it is the person who handles the firearm who is responsible for using it safely, redirecting the onus of safety onto the firearm owner. Further, our participants underscored the need for routine maintenance and proper storage, just as one would need to perform routine maintenance on a car to keep it in good condition.

### Theme 3: Messengers, Modes, and Channels

The data also reveals the importance of the firearm retailer’s role, as a trusted messenger, in educating new and established firearm owners to safely use and maintain their guns. According to participants, this responsibility goes beyond simply selling the firearm; retailers were proud to ensure that customers had access to information surrounding ammunition, cleaning equipment, firearm maintenance, and safe storage and handling practices. Participants also highlighted the immense value of establishing a rapport with customers by demonstrating care for their safety and well-being, and by avoiding an anti-gun stance that might alienate some customers. Moreover, retailers emphasized the importance of human-to-human interaction in educating customers, underscoring the critical role of gun retailers in providing a personalized and empathetic approach to firearm safety education. Finally, retailers generally felt like the most appropriate messengers of firearm safety information and believed they held the requisite expertise to deliver this type of information to gun owners.

Participants were also asked to provide their thoughts surrounding the most effective modes of communicating firearm safety information to patrons. Generally, retailers endorse word-of-mouth and interpersonal counseling as the most effective methods of communicating this information. One retailer specifically stated: “the best ways I’ve seen to having people receive [firearm safety] info is through word-of-mouth and through talking to them on a one-on-one level.” Another retailer implied: “In the retail space I think it’s just an overall conversation, to be honest with you. I don’t have a way to implement [conversations] in a formula every time, because you just have such a vast assortment and range of experiences of people coming into a gun range.” Some retailers did specifically note the lack of structured guidance on conducting firearm safety conversations with potential customers.

Retailers expressed mixed reactions when asked about the utilization of pamphlets to communicate about firearm safety. Some retailers mentioned that “[t]he pamphlet… is convenient, ‘cause it’s something that can be stored with the gun” whereas others report that “[m]ost of the time [pamphlets] end up in the floor or in the trash or they stay in the box at the top of your closet”. Retailers also suggested using visual aids to communicate with audiences pointing to issues of literacy and language barriers among customers. Additionally, participants generally agreed that the use of posters to communicate with patrons about firearms and firearm safety may not be the most effective way to promote firearm safety because “most people don’t stop and read or look at posters or signs” and “people just refuse to look at [them]”. Technology was also suggested as a potential mechanism to communicate firearm safety information — particularly to younger audiences. In particular, retailers believed using mobile apps and social media to communicate to firearm owners represents a next-generation approach for promoting firearm safety among this population.

## Discussion

Findings from this qualitative study offer valuable information related to the communication of firearm safety information from the perspective of firearm retailers – a well-respected and relatively trustworthy group of messengers previously identified in literature. Four audience segments were identified by firearm retailers: new firearm purchasers, gun-owning families, veterans/military personnel, and women. Retailers emphasized the need for tailored messaging to these groups and for the use of language that avoids the demonization of firearms. Analogies are commonly used to normalize firearms, with participants drawing comparisons to common household tools and/or motor vehicles. In addition, the critical role of firearm retailers as credible messengers is implied, with word-of-mouth and interpersonal counseling identified as the most effective means for communicating firearm safety information to patrons in the retail and range environment. Technological delivery channels, like mobile apps and social media platforms, were also discussed as potentially important ways to deliver firearm safety information.

The insights gathered here related to the different audience segments of firearm owners in need of firearm safety information bear a remarkable resemblance to results generated by other scholars in previous studies. Boine et al. (2022) identified two subgroups in a latent class analysis of gun owners that bear a striking resemblance to our results: the first subgroup, characterized by a high percentage of women and motivated by self-protection, is consistent with the firearm-owning women audience segment that emerged in our analysis; the second subgroup, referred to as “the family protectors,” is comparable to the parent and caregiver segment we uncovered in our analysis. However, our study offers a unique contribution to the existing literature by implying that communicators may consider differentiating between newer firearm purchasers and more established firearm owners in their communication efforts as well as tailoring communication efforts to veterans and members of the military population. It is important to note that these segments may have varying media consumption habits, divergent opinions on the credibility of various messengers, different levels of confidence in safely handling a firearm, and may require tailored messaging to effectively communicate firearm safety information. Subsequent research should investigate these intricacies to inform the creation of effective firearm safety promotion strategies.

While this study validated several segments of firearm owners found in prior research, it is important to note the absence of commonly cited groups identified in previous research such as hunters and Second Amendment activists (Boine et al., 2022). There are two potential reasons for this discrepancy. First, the interview guide used in the present study specifically asked retailers about groups of people who might benefit from firearm safety information in the retail setting. In Texas, individuals engaged in hunting are required to complete a hunter’s education course, which may explain why retailers did not readily acknowledge this group as needing additional firearm safety information. Second, retailers were asked specifically whether they tailor safety information based on customers’ appearance and demographics. This line of questioning employed a demographic segmentation strategy rather than a psychographic segmentation strategy (Klöckner & Klöckner, 2015), which may partially explain why groups defined by their ownership motivations did not naturally emerge in the thematic analysis.

In alignment with the conclusions of the current study, a previous investigation into the most effective methods of communicating suicide prevention among firearm owners underscored the fundamental role that language plays in the promotion of firearm safety (Pallin et al., 2019). Notably, both this study and the study completed by Pallin and colleagues (2019) emphasize the importance of substituting the term “weapon” with “firearm,” as the latter conveys a more neutral perception, devoid of any associated connotative, attitudinal, or normative implications. The current research builds upon these initial findings by underlining the need for communicators to avoid “sensationalized” language – which was perceived as being overused by the media and certain political advocacy groups – to foster genuine trust among the firearm owning community. This is particularly relevant, considering previous research that demonstrates certain audience segments among the gun-owning population (i.e., Second Amendment Activists) are likely to express profound distrust in government institutions (Boine et al., 2022; Kelley & Ellison, 2021) and the crucial role trustworthiness has shown to play in persuasion (Milliman & Fugate, 1988), information processing (Trumbo & McComas, 2008), and health communication efforts more broadly (Hocevar et al., 2017).

The study findings here also imply that some firearm owners may possess a less than comprehensive understanding of the intricate anatomy and functionality of the many different firearms available for purchase. This was evidenced by the widespread practice of firearm retailers providing prospective buyers with instruction on the proper terminology for different parts. This initial discovery raises questions and suggests that further exploration is warranted. Specifically, future research could investigate the degree of firearm owners’ knowledge about the firearm’s structural anatomy, as well as their proficiency with its mechanics. Such research could provide a more nuanced understanding of the challenges firearm owners may face when interacting with and using their firearms. It could offer insights into effective strategies to enhance communication regarding the safe and proper handling of firearms. Retailers also frequently commented about the importance of not assuming proficiency with firearm handling and storage among those in professions that require carrying a firearm; this finding has important implications for future research. e.g., one potential avenue for future research is to explore the extent to which individuals in different firearm-related professions receive safety training and education, and whether this training is sufficient to ensure safe firearm handling and storage practices.

Finally, our results suggest that this group of firearm retailers commonly provided personalized firearm safety information in their store, often inquiring about the purpose of the firearm purchase and whether there were children in the household before providing a recommendation. Although several firearm and medical advocacy groups argue that triple safe storage should be implemented in households (i.e., keeping firearms stored locked, unloaded, and away from the ammunition), other qualitative studies have concluded that such blanket recommendations may clash with the urge for self-protection among some gun-owning individuals (Aitken et al., 2020). Our research supports this notion, as we found that retailers frequently consider customers’ self-protection motivations around firearm ownership when engaging in communication with patrons and seem to adapt their safety advice accordingly based on important contextual details that emerge from conversations at the point-of-sale. Future efforts should focus on designing more structured and comprehensive guidance for firearm retailers in what should be assessed and communicated during these conversations.

### Limitations

There are a few limitations that should be noted for the present study. First, the retailers we interviewed were sampled from a single firearm retail shop located in the suburbs of Houston, Texas. Additionally, the majority of the sample we recruited were male and the sample size was relatively small. While this may have limited the generalizability of study findings to other contexts – such as other retail locations that sell firearms (i.e., pawn shops, sporting good shops) and geographies – it is important to acknowledge that the purpose of qualitative inquiry is to provide rich, contextual information about a particular phenomenon rather than to establish generalizability. Through the use of qualitative research methodologies here, our study offers a detailed exploration of the experiences of firearm retail employees in their communication with patrons, providing valuable insights that can inform future research endeavors in other contexts and settings. Moreover, it is crucial to emphasize that the current research is exploratory in nature, and further studies directly involving each of the audience segments identified in this study should be conducted. This future research will be essential to develop a comprehensive understanding of the most effective strategies for tailoring communications to these diverse target audiences. Finally, social desirability bias may have operated in the present investigation. More specifically, participants might have provided responses that they believed the interviewer wanted to hear or that they thought represented the views of their employer, rather than expressing their personal opinions or experiences. Through our informed consent procedure as well as during the interviews, we ensured participants were aware of the steps our team took to protect the privacy of respondents and maintain the confidentiality of their responses, in order to minimize the potential for social desirability bias. Future studies might consider employing more anonymized data collection methods, like self-administered questionnaires, to further minimize the potential for this bias.

## Conclusions

Recognizing the need for more effective communication strategies, this study explored ways to improve firearm safety communication with gun owners by leveraging insights from a relatively credible messenger group, firearm retailers. Overall, this study found that the gun-owning community is composed of distinct audience segments who would benefit significantly from tailored and targeted communication. The findings here stress the need for the development of social marketing efforts that consider the unique psychographic and behavioral characteristics of these various segments as well as the utilization of balanced language that ultimately avoids the demonization of firearms while normalizing their presence through the use of specific analogies. The role of firearm retailers as vital messengers is also highlighted, with interpersonal communication and word-of-mouth counseling during point-of-sale conversations emphasized as particularly efficacious strategies for the dissemination of firearm safety information. Furthermore, our research highlights the need for comprehensive and structured guidance for firearm retailers in their communication efforts, particularly when addressing individuals in professions that require carrying firearms.

In conclusion, this study contributes important insights to the existing literature by leveraging the perspectives of a respected and trusted group of communicators who are already engaging with firearm owners and prospective gun buyers. Furthermore, the convergence of some of the current study’s findings with previous research (Ewell Foster et al., 2024; Pallin et al., 2019) lays a strong foundation for exploring new avenues for message tailoring and audience targeting in this domain. Overall, this work advances our understanding in this critical area and offers promising directions for future health promotion initiatives focused on firearm safety.

## Data Availability

The data that support the findings of this study are available from the corresponding author upon reasonable request.
